# Advancements in acne detection: application of the CenterNet network in smart dermatology

**DOI:** 10.3389/fmed.2024.1344314

**Published:** 2024-03-25

**Authors:** Daojun Zhang, Huanyu Li, Jiajia Shi, Yue Shen, Ling Zhu, Nianze Chen, Zikun Wei, Junwei Lv, Yu Chen, Fei Hao

**Affiliations:** ^1^The Third Affiliated Hospital of Chongqing Medical University, Chongqing, China; ^2^Shanghai Beforteen AI Lab, Shanghai, China; ^3^Simulation of Complex Systems Lab, Department of Human and Engineered Environmental Studies, Graduate School of Frontier Sciences, The University of Tokyo, Tokyo, Japan

**Keywords:** CenterNet network, acne detection, dermatology, deep learning in healthcare, image detection, interpretability

## Abstract

**Introduction:**

Acne detection is critical in dermatology, focusing on quality control of acne imagery, precise segmentation, and grading. Traditional research has been limited, typically concentrating on singular aspects of acne detection.

**Methods:**

We propose a multi-task acne detection method, employing a CenterNet-based training paradigm to develop an advanced detection system. This system collects acne images via smartphones and features multi-task capabilities for detecting image quality and identifying various acne types. It differentiates between noninflammatory acne, papules, pustules, nodules, and provides detailed delineation for cysts and post-acne scars.

**Results:**

The implementation of this multi-task learning-based framework in clinical diagnostics demonstrated an 83% accuracy in lesion categorization, surpassing ResNet18 models by 12%. Furthermore, it achieved a 76% precision in lesion stratification, outperforming dermatologists by 16%.

**Discussion:**

Our framework represents a advancement in acne detection, offering a comprehensive tool for classification, localization, counting, and precise segmentation. It not only enhances the accuracy of remote acne lesion identification by doctors but also clarifies grading logic and criteria, facilitating easier grading judgments.

## 1 Introduction

Acne vulgaris is a prevalent inflammatory skin disease that affects ~9.38% of the global population (1). It can exacerbate negative emotions like anxiety and depression, altering self-perception and potentially affecting social interactions and career paths (2). This comes with a substantial economic toll. For instance, the U.S. spends around $3 billion annually on acne-related treatments (3). Hence, the institutionalization of standardized acne vulgaris therapeutic approaches remains paramount for the betterment of patients and society at large. The impact is especially pronounced in China, this figure reaches 8.1%, with statistics indicating that nearly 95% of individuals will encounter acne at least once (4). Traditionally, the diagnosis of acne has relied on a patient's face-to-face interaction with a dermatologist, but the traditional diagnosis of acne has made it difficult for dermatologists to cope with the increasing demand for diagnosis. Take China as an example, the total number of dermatologists in the country is about 30,000, accounting for only 0.0016% of all acne patients (5). But with the popularity of smartphones, some 1.067 billion Chinese users can easily take and share skin images (6). These devices pave the way for innovative remote dermatological detection applications. At the same time, in order to meet the growing demand for acne diagnosis and improve its accuracy and objectivity, artificial intelligence technology is gradually being introduced into the diagnosis process. Additionally, the COVID-19 pandemic has accelerated the adoption of teledermatology, enhancing acne diagnostics and treatment by enabling continuous patient care in a remote setting (7–9). It is particularly noteworthy that image segmentation, recognition, and grading tasks became central to this technological advance. By analyzing skin images, AI methods can not only accurately diagnose acne, but also provide rapid, objective feedback to patients and healthcare professionals, simplifying interactions and improving the quality and efficiency of care.

In the field of acne detection, image sources and ways of processing have undergone tremendous technological advances over the past few years. Initially, the dermoscope was widely regarded as an effective tool for the initial diagnosis of acne and other skin disorders. In fact, Vestergaard et al. in a 2007 study, verified that dermoscopy can provide a more accurate diagnosis in a clinical setting compared to traditional visual examination (10). However, with the emergence of digital photography technology, high-resolution digital equipment is gradually popular, but its convenience needs to be improved (11). In recent years, due to the increasing popularity of mobile devices, Wang et al. has integrated the entire acne assessment process into mobile devices, allowing smartphone users to assess acne anytime, anywhere (12). In the early stages of automated skin acne detection, the main focus is on how to accurately distinguish between acne and healthy skin. Shen et al. used convolutional neural networks to segment skin and non-skin areas, but large cysts and pustules may not be completely captured at a small resolution (13). To cope with this problem, Zhang and Ma adopted the YOLOv5 model and adjusted the input image size to 224 × 224, which helped reduce information loss and provide a wider field of view (14). However, this model may not work well in cases where acne is densely distributed. In addition, Liu et al. adopted an ensemble learning strategy to improve the specificity and sensitivity of detection but still faced challenges in capturing the subtle features of facial acne vulgaris (15). With the progress of science and technology, the urgent need for accurate technology of acne recognition has gradually become prominent, and a variety of models have been applied to acne recognition tasks. In 2016, fuzzy C-means and support vector machine methods were introduced into acne image recognition (16). Although this approach pushed the field forward early on, the limitations of this type of approach are more obvious than deep learning. Subsequently, Google introduced the EfficientNet-b4 model, which showed excellent performance by integrating multiple types of information for acne diagnosis, but the interpretability of its model still needs to be further studied and optimized (17). In 2023, Josphineleela conducted dermatological classification based on Faster RCNN and achieved good detection efficiency and excellent performance in dermatological classification (18). At the same time, a new model combining YOLOv5 and ResNet50 was used for the detection of skin pigmentary lesions, which was superior to the existing technology in depth feature extraction (19). In the field of acne severity grading research. Yang et al. provides acne assessment and treatment recommendations based on current acne management guidelines in China, but do not classify and quantify specific skin lesions (20). Lin's team proposed a global assessment grading framework simulating dermatologist diagnosis to adapt to various grading criteria (21). In 2022, a study used the Global Assessment (IGA) scale to grade the severity of acne, with counts of various acne types as inputs, and the model was 6.8% more accurate than physician diagnoses (22). Overall, the automated skin acne detection field has evolved significantly, transitioning from initial explorations to advanced technological frameworks. This progression has enhanced acne diagnostic technologies and established a foundation for greater diagnostic precision and efficiency. Despite these advancements, current methods face challenges, particularly in high-acne-density scenarios where models like convolutional neural networks and YOLOv5 struggle with low-resolution detail capture. Additionally, while models like EfficientNet-b4 consolidate diverse diagnostic data, their interpretive capacity is limited, hindering broader clinical application. Addressing these issues, namely enhancing image resolution, information integration, and model interpretability, remains pivotal in ongoing research.

In previous experiments, it demonstrated a high recognition rate for acne (75.98%) through extensive testing on 33 different facial skin diseases, demonstrating its accuracy in skin disease recognition. Therefore, this article on the basis of more in-depth acne type identification (23). Our study developed an acne detection model that harnesses the power of the CenterNet network, realized through a collaborative effort between dermatologists and computer science researchers. This model is designed to automatically analyze facial images taken with smartphones, serving as an invaluable adjunct tool for dermatologists: 1. The image quality can be identified on the mobile terminal, and conditions such as image light and face occlusion can be automatically identified to meet the requirements. 2. The center point estimation method can be used. The location of the target was determined by predicting the center of the target, so that the network could better integrate the global and local information. 3. It can not only obtain accurate classification results, but also clearly explain the judgment criteria of classification. The data used in this study consisted of images collected by smartphones and clinical images collected by clinical hospitals, which were subsequently labeled by recruited dermatologists and used for AI training. Overall, this model not only improves the accuracy and efficiency of diagnosis, but also provides solid technical support for clinical application while ensuring the transparency and interpretability of the decision.

## 2 Methods

### 2.1 Image data processing

#### 2.1.1 Data source and collection

To train and validate our algorithm, we collected 212,374 original acne lesion images from dermatology clinical clinics in 15 hospitals in different provinces of China using smartphones from January 2020 to October 2022. For assessing the suitability of each image, we employed Google's advanced machine vision technology, Mediapipe, which evaluates images based on clarity and exposure. Firstly, the face region of the image is cropped and six feature values of the cropped image are calculated, and the sharpness is predicted by logistic regression model. Then, the image was converted from RGB three channels to H(hue)S(saturation)V(lightness), and according to the pixel value of V channel, whether the image was too dark or over-exposed was judged. Finally, based on these criteria, we excluded occluded lesions, non-face images, overexposed, underlit, and blurred images. Through image usability testing and inclusion criteria, 153,183 acne lesions images were finally selected, from which a training set containing 150,219 images and a validation set containing 2,322 acne lesions images were constructed for subsequent modeling.

#### 2.1.2 Data annotation and review

In the process of labeling the collected facial acne images, in order to label the lesion types efficiently and accurately, we design annotation rules. First, for relatively regular and uniform size acne lesions, such as comedones, papules, pustules, and nodules, we use rectangular boxes for labeling. However, for lesions with more complex and irregular morphology, such as cysts and scars, we choose to use polygonal boxes for annotation. In this way, we can ensure that no critical morphological information is missed during the data annotation process, thus increasing the diagnostic accuracy of the model. To ensure the accuracy and consistency of labeling, all participants received standardized training before the start of the study to ensure that they were proficient in operating the assessment tool and had a thorough understanding of the classification criteria for acne lesions. In addition, the labeling results were reviewed by two dermatologists. These two experts will correct any labeling errors and omissions to ensure the accuracy of the data. When there are differences of opinion among experts, they will reach consensus through discussion.

### 2.2 Acne detection module

#### 2.2.1 CenterNet architecture

Our proposed acne detection algorithm is based on the CenterNet target detection framework and aims to accurately locate acne lesions and classify their types. CenterNet uses anchor-free detection to directly predict the center point, width and height of the target, enabling efficient target location and identification (19). This method avoids matching problems that may be caused by fixed size and proportion of anchor frames in traditional target detection algorithms. In addition, due to the small size and irregular shape of acne lesions, predefined anchor boxes are less effective, while CenterNet's frame-free approach eliminates the complexity of finding the right anchor box, providing a distinct advantage for acne detection (24). In view of the many clinical forms of acne, such as comedones, papules, pustules and nodules, especially cysts and scars that are irregular in shape and vary in size, we have optimized CenterNet's infrastructure by integrating a specialized submodule for segmentating cysts and scars, enhancing the model's credibility and transparency in the medical diagnostic process. Finally, we mapped the model results to the rating criteria of China Acne Treatment Guidelines (2019), and compared the performance of the model with that of the most popular models, Faster RCNN, RetinaNet, YOLOV3, YOLOX, and EfficientDet.

At the heart of CenterNet lies its adoption of the deep hierarchical aggregation (DLA) strategy, utilizing DLA34 as the foundational feature extraction network. Within DLA34, two pivotal modules, namely iterative depth aggregation (IDA) and hierarchical depth aggregation (HDA), play a crucial role. They effectively amalgamate feature information across various levels. Firstly, DLA34 enhances the model's object recognition capability through semantic aggregation. This enhancement is realized by fusing feature information in the channel directions, equipping the model with a superior understanding of the semantic information present in acne images. As a result, the model becomes adept at identifying and distinguishing between various types of acne, cysts, and scars. Secondly, DLA34 incorporates spatial fusion, which includes the use of deformable convolutions. These convolutions introduce orientation parameters for each element, broadening their coverage during training. This ensures a more thorough analysis of images, especially when addressing irregularly shaped cysts and scars. Such features empower the model to precisely identify, locate, and segment different acne lesions, offering robust support for multi-classification tasks.

#### 2.2.2 Loss function

In our optimization of the CenterNet network, the model needs to handle object detection and semantic segmentation tasks in parallel. This requires us to fuse the loss function of the two task detection loss*L*_*det*_ and the segmentation loss *L*_*seg*_:


$$L_{total}=L_{det}+L_{seg}$$

*L*_*det*_ consists of the center point prediction loss *L*_*k*_, the target center bias loss *L*_*off*_, and the target size loss *L*_*size*_, which is calculated as follows:


$$L_{det}=L_{K}+\lambda_{size}L_{size}+\lambda_{off}L_{off}$$

Where λ_*size*_=0.1λ_*off*_=1. The center point prediction loss *L*_*k*_ is usually calculated using the binary cross-entropy loss. This loss measures the difference between the predicted target center heat map and the true target center heat map, and *L*_*k*_ is calculated as follows:


$$L_{k}=-\frac{1}{N}\sum_{ryc}\left\{ \begin{array}{l} {\left(1-{\hat{Y}}_{ryc}\right)}^{\alpha }\log\left({\hat{Y}}_{ryc}\right)\text{ if }Y_{ryc}=1\\ {\left(1-Y_{ryc}\right)}^{\beta }{\hat{Y}}_{ryc}^{\alpha }\\ \log\left(1-{\hat{Y}}_{ryc}\right)\text{ otherwise} \end{array}\right.$$

Where α and β is the hyperparameter of Focal Loss and N is the number of pixels of the feature map after downsampling.

Since the model predicts the center point on the low-resolution feature map, a small bias is also predicted in order to improve the localization accuracy. The loss in this part is usually calculated using the L1 loss. The bias loss Loff of the target center is calculated as follows:


$$L_{\text{off}}=\frac{1}{N}\underset{p}{\sum }|{Ô}_{p}-\left(\frac{P}{R}-\tilde{P}\right)|$$

Where *O*_*p*_ is the predicted bias and $\frac{P}{R}-\tilde{P}$ is the actual error computed in advance during training.

*L*_*size*_ uses L1 loss to compute the difference between the predicted and true object sizes, and regress each object size as follows:


$$L_{\text{size}}=-\frac{1}{N}\overset{N}{\underset{k=1}{\sum }}|{Ŝ}_{k}-{\tilde{S}}_{k}|$$

Segmentation loss *L*_*seg*_ uses L2 loss to calculate the error of cysts and scars segmentation results, which is calculated as follows:


$$L_{\text{seg}}=\frac{\overset{N}{\underset{i=1}{\sum }}{\left(I_{i}-{Ĩ}_{i}\right)}^{2}}{N}$$

Where I is the segmentation prediction result, Ĩ_*i*_ is the segmentation ground truth, and N is the number of pixels of the segmentation prediction result.

### 2.3 Test suite construction

In order to further evaluate the effect of our model in real-world clinical application, we collected the data in the clinical outpatient department of dermatology of a Class Grade A hospital as an independent test set. Following medical ethical guidelines, all participants were required to sign two informed consent forms in advance. We performed the relevant image acquisition only after obtaining the explicit consent of the patient. To ensure the integrity and reliability of the study data, a clear frontal image of each patient was taken using a smartphone.

Patients were admitted to this study only if they met all the inclusion criteria. The inclusion criteria of patients in the module were: 1. The age was not < 18 years old; 2. Current diagnosis of facial acne, but not other facial diseases such as fulminating acne, acne conglobata, folliculitis, and rosacea. 1. The image must be a clear mug shot, and the shooting Angle, distance and light must be within the specified range; 2. The face area of the image should be clear, without occlusion, and can be accurately cropped and identified; 3. The color of the image should be true without excessive color deviation.

**Figure 1:**
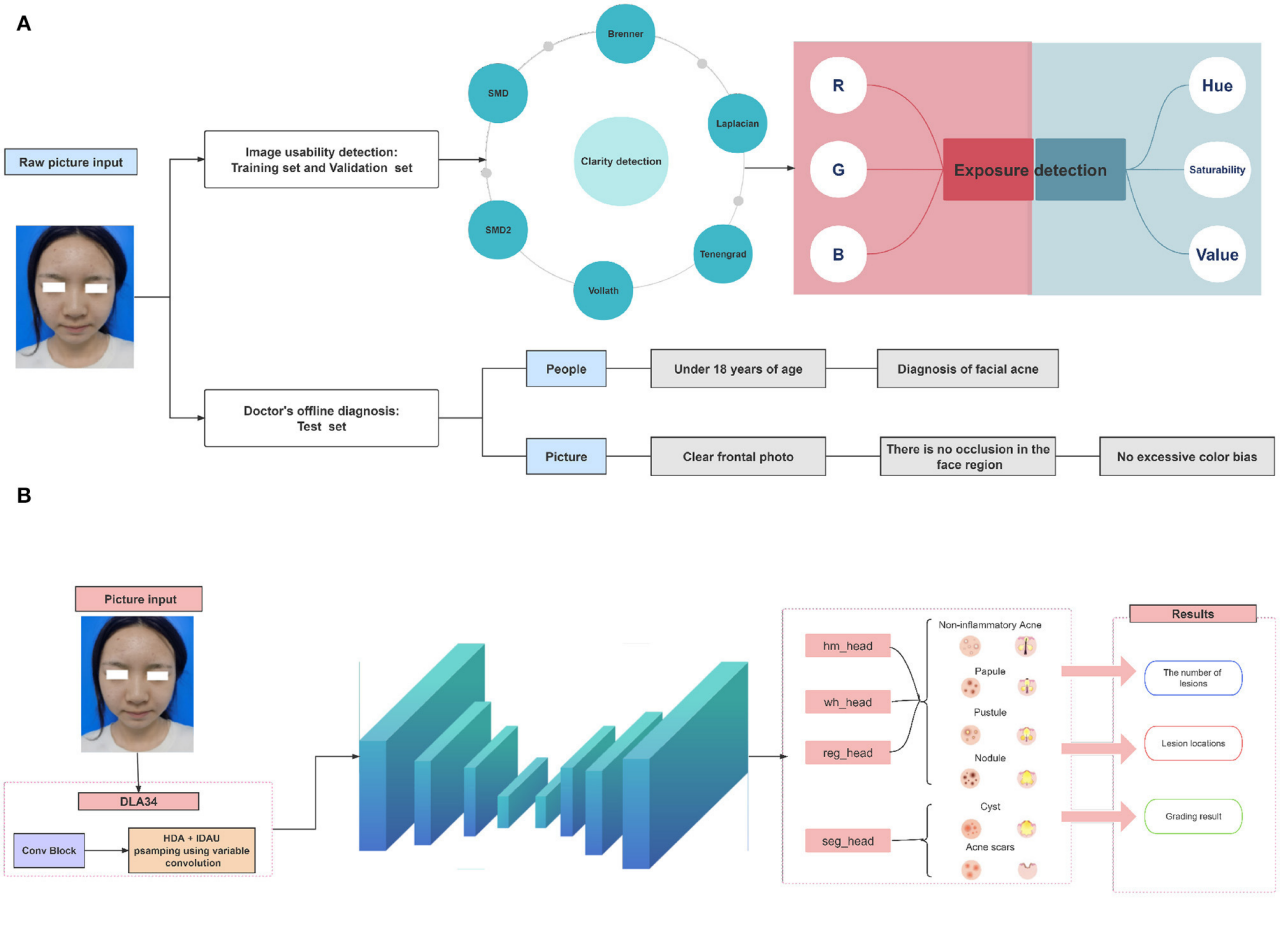
Overall framework of the acne detection algorithm. **(A)** We obtained 212,374 maps of original acne lesions. According to the required conditions, 153,183 images were obtained, of which 150,219 were training sets, 2,322 were validation sets and 642 were test sets. **(B)** Facial images are collected as input data, and a deep hierarchical aggregation strategy is used to effectively fuse different levels of feature information to create a classification module for identifying comedones, papules, pustules, and nodules, and a segmentation module for identifying cysts and acne scars. Finally, according to the recognition results, the acne grading results were output.

In total, we collected 642 images at this stage. To ensure the accuracy of the test set, we invited a senior dermatologist with more than 15 years of clinical experience to perform offline diagnosis and to review the results online with another senior physician. In addition, in order to evaluate the diagnostic effect of the model and dermatologists, we also collected the diagnostic results of five middle and low seniority dermatologists as a comparison benchmark (Figure 1).

In order to verify the performance of the acne detection model, we adopted the grading method of the Chinese Acne Treatment Guideline (2019 revision). According to the guidelines, the severity of acne can be divided into third degree and fourth degree according to the type of lesion. The grading criteria are as follows: 1. mild (grade I) : only comedones. 2. moderate (grade II) : papule. 3. moderate (grade III): pustule. 4. severe (grade IV): cyst and nodule. Furthermore, we calculated the consistency of the results of the Kappa coefficient comparison grading to evaluate the consistency of the acne detection model and between dermatologists at all levels and the reference gold standard, and further verified the comparability and interpretability of the model.

### 2.4 Model performance indicators

In this study, we focused on several main evaluation indicators: Precision, Recall, error, F1 score, and AP (Average Precision) under the precision-recall curve. The precision rate refers to the proportion of all samples predicted to be positive cases, which are actually positive cases. Recall is also called recall, which is for the original sample, and its meaning is the probability of being predicted to be a positive sample in the actual positive sample. The range of average precision AP was usually between 0 and 1, and the closer to 1, the better balance between accuracy and recall was achieved. The F1 value is the harmonic average of Precision and Recall, which takes into account both precision and coverage. In addition, to further validate the performance advantages of the models, we compared them with models such as Faster RCNN, RetinaNet, YOLOV3, YOLOX, and EfficientDet.

## 3 Result

### 3.1 Overview of approach

In this comprehensive study, we rigorously evaluated the performance of our advanced acne detection model. The dataset, consisting of 150,219 training images, 2,322 validation images, and 642 test images, offered a robust platform for analysis. Our focus was on the model's capability to accurately detect and classify various acne types, leveraging state-of-the-art statistical methodologies for a thorough evaluation. The data distribution of lesions in the overall sample of our images is shown in Table 1.

**Table 1:** Overall sample image lesion situation table.

**Average acne per picture**	**Comedones**	**Papule**	**Pustule**	**Nodule**	**Cyst**	**Scar**
Test set	3.70 (46.27%)	1.37 (17.14%)	0.16 (1.99%)	0.03 (0.35%)	0.27 (3.39%)	2.47 (30.86%)
Validation set	4.89 (37.04%)	2.74 (20.74%)	0.35 (2.69%)	0.09 (0.68%)	1.03 (7.84%)	4.09 (31.01%)
Training set	3.56 (40.18%)	2.42 (27.31%)	0.14 (1.63%)	0.10 (1.10%)	0.23 (2.62%)	0.23 (2.62%)

The data set in this study was designed to reflect the clinical incidence and diversity of acne lesions. The distribution of lesions in the training set was characterized by a high prevalence of comedones, papule and scar (40.18, 27.31, and 27.16%, respectively, in the dataset), which was consistent with the commonality of diseases in the clinical environment. The incidence of pustules, nodules, and cysts is low, but the incidence of this type of acne is low in the general patient population. The validation set reflected the distribution of the training set, with slightly higher incidences of cysts and scars, which accounted for 7.84 and 31.01% of cases, respectively. This adjustment ensures that the validation of the model is robust for a wider range of lesion types. In contrast, the test set presented a different Acne distribution, with a greater proportion of comedones (46.27%) and a reduced prevalence of nodules and pustules. This variance is instrumental in assessing the model's adaptability and performance across different clinical settings. The composition of the test set encompasses the multifaceted nature of acne and validates the breadth of manifestations that acne detection models may encounter in real-world clinical Settings. In summary, the composition of these data sets fully considers the various situations that acne detection models may encounter in practical clinical applications, ensuring the generalization ability and accuracy of the models.

### 3.2 Performance analysis of acne detection model

**Figure 2:**
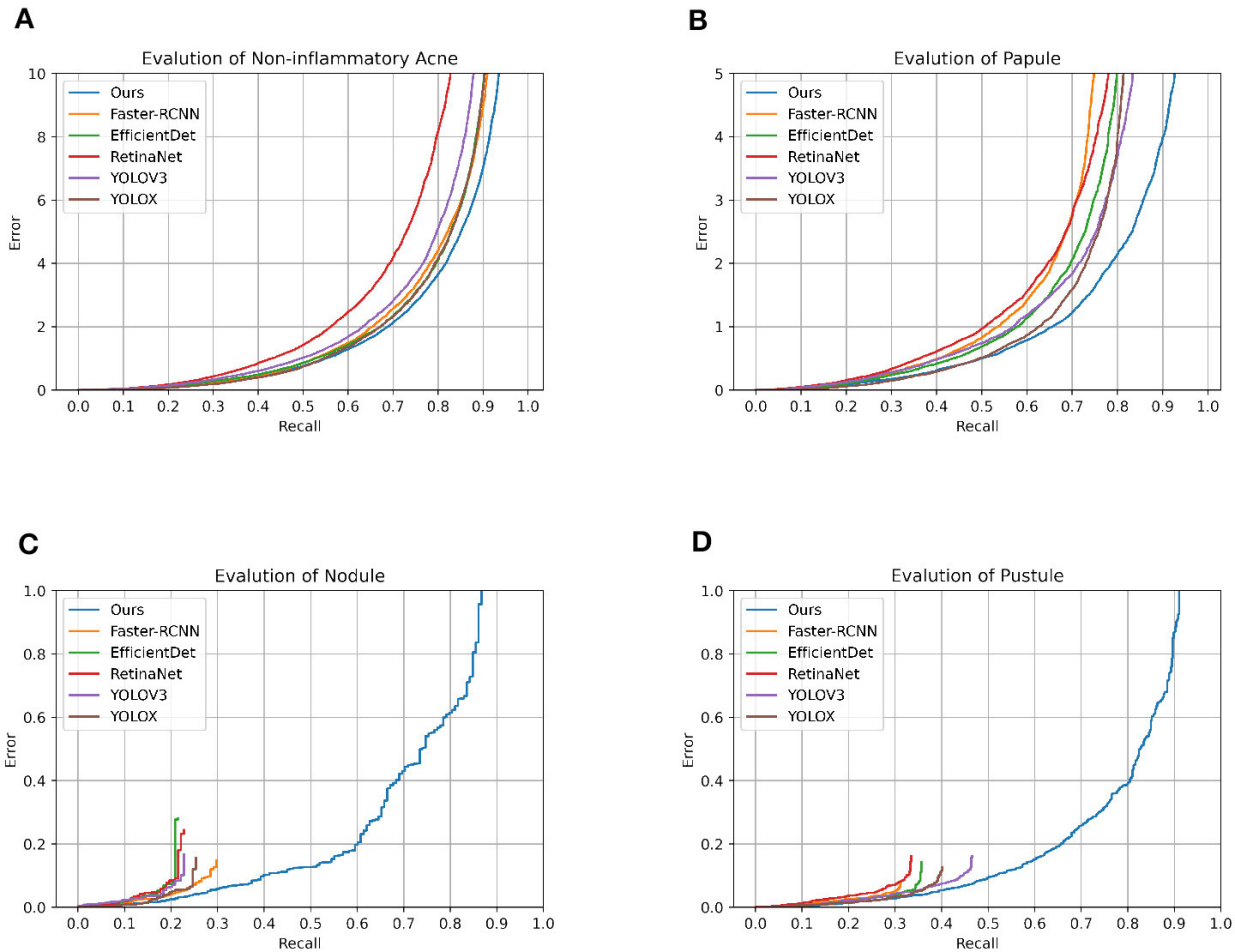
Model error_recall graph. This is the error_recall plot of the various models on different classifications.

In the realm of acne lesion detection, CenterNet asserts its clinical utility through the lens of rigorous quantitative measures. Against the backdrop of established models, CenterNet emerges with a kappa statistic of 0.833, denoting a substantial concordance with clinical diagnosis, surpassing the 0.642 of Faster RCNN. While Faster RCNN demonstrates high accuracy, particularly with nodules at 91.4%, its F1 scores for pustules at 60.3% and nodules at 30.3% reveal a diminished precision-recall balance, hinting at the model's nuanced challenges in delineating complex lesion types. RetinaNet offers a consistent detection capability across lesion types, yet its performance does not eclipse that of CenterNet, which achieves a more harmonious accuracy-recall equilibrium, particularly reflected in a superior F1 score for nodules at 33.9% compared to RetinaNet's 26.5%. Although YOLOV3 achieved 92.8% accuracy for nodules, it ran into obstacles in terms of clinical consistency, with a kappa statistic of 0.608, which did not meet the benchmark set by CenterNet. Similarly, YOLOX parallels Faster RCNN's accuracy for comedones but falls short of CenterNet's consistency, highlighted by a kappa statistic equal to Faster RCNN's at 0.64. EfficientDet competes closely in terms of accuracy, but a kappa statistic of 0.602 indicates a potential for enhancement in its clinical correlation. Moreover, the accuracy of CenterNet for comedones, at 83.8%, is slightly higher than that of RetinaNet at 81.2%. And the kappa statistic demonstrates a significant advancement, indicating that CenterNet is more finely tuned to the intricacies of clinical diagnosis. These comparative findings not only validate the robust diagnostic capabilities of CenterNet but also highlight its potential for seamless integration into clinical workflows. The data underscores the necessity for adaptive and precise detection methodologies, positioning CenterNet as a frontrunner in terms of diagnostic accuracy and alignment with clinical standards (Figure 2).

CenterNet's prowess in automated acne lesion detection is characterized by its high accuracy and precision. In categorizing comedones, CenterNet achieved a max_recall of 0.985 and an AP of 0.625, outperforming the second best model's max_recall of 0.833 by a notable margin. Its superior performance extends to the identification of papules and pustules, with max_recall values exceeding 0.9, demonstrating its proficiency in discerning lesions with variable shapes and pigmentation. The model's adeptness in differentiating between acne types is corroborated by F1 scores of 0.888 for comedones and 0.858 for papules. These scores not only stand out in the context of classification categories but also signify the highest performance against competing models. For pustules and nodules, CenterNet's F1 scores, while not the highest across all types, represent the peak within this specific model comparison, emphasizing its potential and identifying areas for targeted refinement in the center point detection algorithm, especially for smaller or less defined lesions. The robustness of CenterNet is evident in its handling of pustules and nodules. It surpasses competing models, achieving an F1 score of 0.659 for pustules—significantly exceeding the average F1 score of 0.60 recorded by other models. This superior detection capability is consistent across varying sample sizes and is reinforced by steadfast max_recall rates for the more commonly presented acne types, affirming its clinical applicability. While there is an opportunity to enhance the model's precision for certain lesion types, CenterNet's overall functionality exhibits an advanced level of clinical alignment (Table 2).

**Table 2:** Performance table for each model inspection.

**Models**	**Index**	**Comedones**	**Papule**	**Pustule**	**Nodule**	**Single image Acc**	**Single case kappa**
Faster RCNN	Acc	0.833	0.796	0.918	0.914	0.510	0.642
	F1	0.886	0.839	0.603	0.303		
RetinaNet	Acc	0.812	0.783	0.902	0.917	0.477	0.640
	F1	0.871	0.835	0.557	0.265		
YOLOV3	Acc	0.823	0.798	0.914	0.928	0.492	0.608
	F1	0.877	0.840	0.639	0.282		
YOLOX	Acc	0.833	0.830	0.921	0.912	0.54	0.64
	F1	0.883	0.862	0.623	0.295		
EfficientDet	Acc	0.817	0.809	0.920	0.918	0.499	0.602
	F1	0.875	0.850	0.608	0.237		
CenterNet	Acc	0.838	0.819	0.921	0.917	0.626	0.833
	F1	0.888	0.858	0.659	0.339		

### 3.3 Comparison of classification abilities between dermatologists and AI

**Figure 3:**
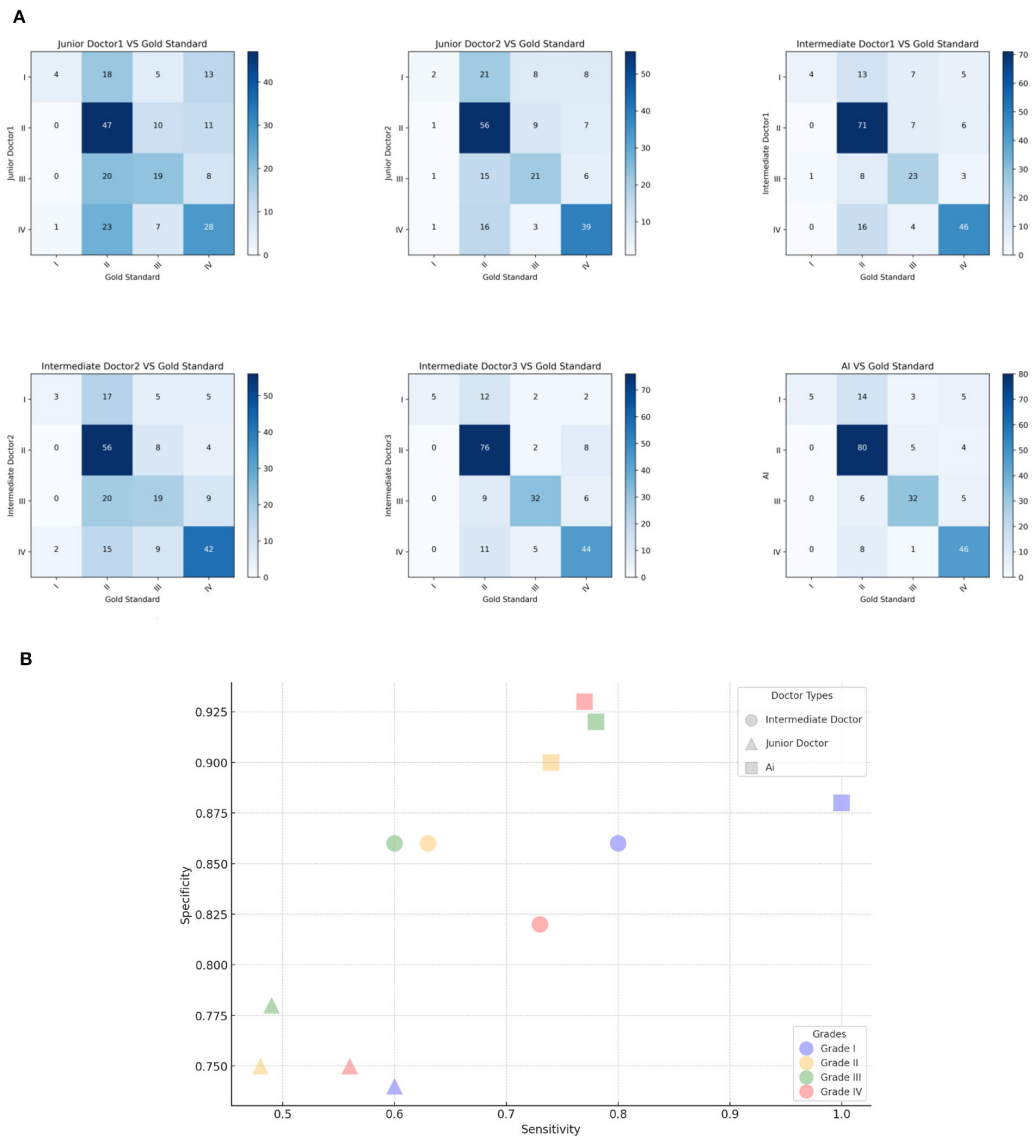
Model classification diagram. **(A)** The picture shows the confusion matrix of Intermediate dermatologist1, Intermediate dermatologist2, Intermediate dermatologist3, Junior dermatologist1, Junior dermatologist2, and AI. **(B)** Sensitivity and Specificity analysis. The *x*-axis is the Sensitivity value, the *y*-axis is the Specificity value.

In this clinical study, confusion matrix data were analyzed to evaluate the diagnostic accuracy of an AI model compared to dermatologists at varying levels of experience in grading acne. The analysis revealed that the AI model achieved an overall accuracy rate of 76%, outperforming both intermediate (65.3%) and junior dermatologists (50.3%). Specificity values further underscored this difference, with junior dermatologists averaging 0.75, intermediates 0.85, and the AI model at 0.91, highlighting its consistent ability to accurately identify true acne cases. The AI model demonstrated a tendency to downgrade severe acne cases, with 70.59% of its misdiagnoses categorized as milder conditions. In contrast, junior and intermediate dermatologists showed more instability classification, with downgrade misdiagnoses at 58.49 and 54.79%, respectively. Cross-grade misdiagnosis was most frequent among junior dermatologists (18.26%), nearly double that of the AI model (9.35%), and intermediate dermatologists exhibited a rate of 13.83%. Both junior and intermediate dermatologists displayed instability in their diagnostic outcomes, especially evident in their uniform misdiagnosis distribution across Grades 2 and 3. Their lower sensitivity for these grades (junior: 0.48 and 0.49; intermediate: 0.60 and 0.63; AI: 0.74 and 0.78) indicated challenges in accurately diagnosing intermediate stages of acne. For Grade 4 acne, junior dermatologists demonstrated a sensitivity of 0.56 and specificity of 0.75, posing a 44% risk of missing actual cases. This was compounded by their tendency to misclassify severe acne as milder, such as Grade 1. In contrast, the AI model showed high sensitivity (0.77) and specificity (0.93) in diagnosing Grade IV acne, with its potential in assisting junior and intermediate dermatologists. This study further introduced Kappa coefficient as an index to measure diagnostic consistency and considered the possibility of random consistency. The AI model is highly consistent with the acne grading standard (kappa value 0.648), and has high accuracy and reliability in the identification and classification of different acne grades. Dermatologists with different levels of experience showed significant differences in consistency. Compared with junior dermatologists (two junior dermatologists with kappa values of 0.243 and 0.366, respectively), intermediate dermatologists (three intermediate dermatologists with kappa values of 0.520, 0.376, and 0.605, respectively) showed some improvement. This observed instability or grading ambiguity among human dermatologists suggests a lack of consistent diagnostic approach in categorizing acne severity, pointing to a broader range of uncertainty in their clinical judgment. The AI models have demonstrated greater stability and accuracy in several instances, yielding superior results in challenging diagnostic scenarios (Figure 3).

## 4 Discussion

This study proposes and implements an acne detection and grading system based on machine learning. The system is specifically designed to parse facial images taken by smartphones and provide physicians with an accurate assessment of the number of acne cases and their severity level. This method performs well in reducing the human error inherent in traditional manual counting, providing an efficient and more accurate solution for acne grading. Compared with previous diagnostic methods that rely on intuitive judgment, our model significantly improves the quantification and objectivity of diagnosis. In the design of the algorithm, we paid special attention to transforming the diagnostic process of the “black box” model into a system with high transparency and interpretability. The model not only outputs results, but more importantly, it provides a visual explanation of the decision-making process, ensuring that the physician can clearly understand the reasoning logic of the model. This transformation allows physicians to accurately determine the nature and severity of the lesion, effectively track the evolution of the lesion during treatment, and adjust the treatment plan at the appropriate time.

In this study, compared with data collection methods that rely on professional cameras or pre-built databases (13, 25), data collected by smart phones can provide more real-time and diversified images of clinical cases. The innovation of this approach is that it ADAPTS to the variability that is common in everyday clinical Settings, increasing the model's ability to process real-world data. Second, we designed the image quality inspection module to set high standards for data preparation, ensuring that the model is trained using high-quality data. This step is critical to improving the performance of the model, as in complex clinical image processing, image sharpness and lighting conditions greatly affect the final result. In terms of target detection, the CenterNet model adopted in this study showed higher accuracy than the traditional model in identifying irregular lesions, especially in pixel-level segmentation. By predicting the central point of an object and generating a heat map for a specific category, CenterNet is able to efficiently locate acne of varying sizes. This technology breaks through the limitation of traditional models such as Faster RCNN and RetinaNet, which rely on candidate box generation strategy to identify the approximate location of the target, and provides a more sensitive and accurate detection method for irregular targets (26, 27). Therefore, the model can well introduce segmentation branches to distinguish between common acne types and irregular acne types. The results show that our model achieves 0.76 accuracy on average, compared to 0.67 accuracy in existing studies such as Ziying Vanessa and her team (5). Finally, in terms of acne grading accuracy, the model showed a high degree of agreement with senior physician ratings, especially in the identification of grade III and IV acne, which reduced the possibility of misjudgment caused by the subjective experience of the physician. This study not only advances the state of the art in acne detection and grading, but also highlights the feasibility of using non-specialist camera equipment in clinical practice. This innovative research method and technology application is of great significance for the future development of the field of medical image analysis, and is expected to provide auxiliary tools in daily clinical diagnosis and improve the accuracy and efficiency of disease diagnosis. We expect that this system can be further improved and widely used in clinical practice in the future.

In this study, we demonstrate the potential of a CenterNet-based acne detection model. While some progress has been made, several key limitations remain to be noted. First, current methods do not take into account the differences in the importance of acne in different areas of the face, which may have an impact on the accuracy of treatment decisions and condition assessments. Second, the model has not been extended to the diagnosis of acne in the trunk of the body, an area that is equally important in clinical practice. Finally, although the acne grading criteria adopted provide a basic framework for the experiment, there is still room for further improvement and optimization given the diversity of acne types and the complexity of clinical practice. Future studies should explore these limitations to more fully evaluate the potential of AI in acne diagnosis and treatment.

In future studies, a number of innovative measures will be taken to enhance our acne detection system. Recognizing that our current model, based on feature extraction from single images, may not suffice for all grading standards, we plan to refine this approach. This improvement will focus on adapting the model to accommodate a broader range of diagnostic criteria, ensuring a more comprehensive and accurate assessment of acne severity from multiple image perspectives. Secondly, when analyzing the experimental results, it was noted that nodular and pustular acne had a small sample size in the existing dataset. In order to solve this limitation, the study is expected to adopt advanced data enhancement techniques, such as rotation, flip, scaling, etc., to artificially expand the dataset of rare acne types, in order to enhance the model's learning and recognition ability of these acne types. In addition, considering that the ruptured and unruptured status of acne has an important impact on patient treatment choice and clinical outcome, a new classification dimension will be introduced to improve the fineness of the classification. Further, given that acne's ulceration status has a decisive impact on condition assessment and treatment options, the team will work to introduce new categorical dimensions aimed at more precisely grading acne to provide patients with more refined diagnostic information. Considering the potential of GAN (SLA-StyleGAN) (28) and whole-body photography (TBP) (29) technologies to improve model performance, future research will focus on evaluating the practical application value of these innovative technologies to further refine our acne detection system.

## Data availability statement

The raw data supporting the conclusions of this article will be made available by the authors, without undue reservation.

## Ethics statement

The studies involving humans were approved by Ethics Committee of the Third Affiliated Hospital of Chongqing Medical University (Jer Hospital). The studies were conducted in accordance with the local legislation and institutional requirements. The participants provided their written informed consent to participate in this study. Written informed consent was obtained from the individual(s) for the publication of any potentially identifiable images or data included in this article.

## Author contributions

DZ: Supervision, Resources, Project administration, Methodology, Investigation, Funding acquisition, Data curation, Conceptualization, Writing—original draft. HL: Supervision, Resources, Project administration, Methodology, Investigation, Funding acquisition, Data curation, Conceptualization, Writing—original draft. JS: Visualization, Methodology, Investigation, Formal analysis, Data curation, Writing—original draft. YS: Visualization, Validation, Project administration, Methodology, Writing—review & editing. LZ: Software, Methodology, Data curation, Writing—review & editing. NC: Visualization, Validation, Investigation, Data curation, Writing—review & editing. ZW: Validation, Software, Methodology, Writing—review & editing. JL: Visualization, Validation, Supervision, Project administration, Investigation, Formal analysis, Data curation, Writing—review & editing. YC: Project administration, Data curation, Writing—review & editing. FH: Validation, Supervision, Project administration, Investigation, Writing—review & editing.
